# Linear energy transfer-independent calibration of radiochromic film for carbon-ion beams

**DOI:** 10.1016/j.phro.2022.08.001

**Published:** 2022-08-08

**Authors:** Mutsumi Tashiro, Motohiro Kawashima

**Affiliations:** Gunma University Heavy Ion Medical Center, 3-39-22 Showa-Machi, Maebashi, Gunma 371-8511, Japan

**Keywords:** Carbon-ion beam therapy, Film dosimetry in spacer, LET-independent simple calibration, Radiochromic film, Gafchromic RTQA2 film

## Abstract

For carbon-ion beams, radiochromic film response depends on the dose and linear energy transfer (LET). For film dosimetry, we developed an LET-independent simple calibration method for a radiochromic film for specific therapeutic carbon-ion beams. The measured film doses were calibrated with a linear function within 5% error. The penumbra positions of the films were consistent with the differences from the planned ones within ~0.4 mm. The results indicated sufficient accuracy for use as a tool for the confirmation of the penumbra position of the fields.

## 1 Introduction

In radiation therapy, confirmation of the doses and field shapes of irradiations is essential for quality assurance (QA). Radiochromic films are convenient tools widely used for QA measurements [1]. However, the film response of particle beams depends not only on the dose but also on the linear energy transfer (LET) [2], [3], [4], [5], [6], [7], [8]. Regarding the LET dependence known as LET quenching [9], above 40% variations in film responses have been reported in the LET range of clinical carbon-ion beams [3]. Therefore, for accurate dosimetry of carbon-ion beams, it is necessary to perform detailed calibration and determine the LET at each point irradiated on the film in advance. However, determining LET distributions in actual treatment beams is problematic.

Carbon-ion beam therapy is advantageous in that it achieves a sharp dose distribution by utilizing the Bragg peak, less multiple scattering, and greater biological effectiveness compared with those of other therapeutic radiations. However, if the organ at risk (OAR), such as the gastrointestinal tract, is adjacent to the target, we are often forced to deliver inadequate doses to the target. Inserting a spacer between the target and the OAR is an effective method to determine the potential of carbon-ion beam therapy [10]. The dose reduction in the OAR can be confirmed by inserting a radiochromic film into the spacer in actual therapeutic irradiations. Furthermore, because the beams used in the treatment of a specific disease follow a prescribed clinical protocol, the prescribed dose is restricted; moreover, the range of the LET of the mixed beam is also limited. If a simple calibration method without the need to determine LET can be devised for such limited doses and LETs, it will be easier to measure the doses or field shapes of the carbon-ion beams with films in patients where the actual LET determination is difficult.

No existing study has investigated the LET dependence of carbon-ion beams for a model of radiochromic films that is mainly used for the irradiation field shape confirmation. Therefore, this study aimed to establish an LET-independent simple dose calibration method for the radiochromic film for specific therapeutic carbon-ion beams, as well as evaluate the accuracy of the dose and the irradiated field edge position.

## 2 Materials and methods

### 2.1 Beam conditions and treatment planning

Several beam conditions were selected assuming carbon-ion radiotherapy irradiation. Three beam energies (400, 380, and 290 MeV/u) and the sizes of the spread-out Bragg peak (SOBP) (90, 60, and 30 mm) clinically feasible at our facility were selected. For these beams, the treatment plans were made with a virtual water phantom for film irradiation, and the dose distributions were obtained for a rectangular irradiation field (multi-leaf collimator opening: 65.2 mm × 67.5 mm) using an XiO-N treatment planning system (TPS) (Elekta, Stockholm, Sweden) (grid size: 2 mm) [11], [12]. The prescribed clinical dose (relative biological effectiveness (RBE)-weighted dose) was set as 4.8 Gy (RBE) based on the cervical cancer protocol [13], [14]. The physical dose varied in the SOBP with a constant clinical dose because the dose-averaged LET and, consequently, RBE changed depending on the depth [15], [16]. The prescribed physical dose was defined as that at the center of SOBP for each beam. Hereafter, “dose” refers to “physical dose” unless specified otherwise.

### 2.2 Film irradiation

Gafchromic RTQA2 films (size: 10 in. × 10 in.) (Ashland, Covington, Kentucky, USA) were cut to the quarter size. The depth of the film was adjusted using a tough water phantom board (PH-40, Kyoto Kagaku Co., ltd., Kyoto, Japan). The film was placed at the desired depth in the stacked phantom boards, and the carbon-ion beam irradiation was applied. The experiments were conducted at the Gunma University Heavy Ion Medical Center (GHMC) with a passive carbon-ion beam port of a vertical direction [17], [18]. The depth of the SOBP center was set to the isocenter plane. Because the main objective was to confirm the dose reduction of OAR close to the target in the expected clinical practice, the measurement depths were selected in the SOBPs as follows:

Beam 1: A:159, B:179, C:199, D:219, E:239 mm.

Beam 2: F:176, G:196, H:216 mm.

Beam 3: I:124 mm.

The stopping power ratio of the carbon-ion beam in the tough water phantom was estimated to be 1.01, and the water equivalent depths were corrected when compared with the planned dose.

### 2.3 Dose measurements by an ionization chamber

Dose distribution measurements were performed using a PinPoint ionization chamber (PTW model 31014) at the same depth as the film measurements. Lateral dose distributions were obtained by scanning the chamber in the longitudinal direction by moving the couch. The standard deviation was estimated to be approximately 0.33% on average from five repeated measurements.

### 2.4 Film scan and analysis

The film was scanned at 16-bit monochrome with a resolution of 150 dpi using a scanner (ES-10000G, Seiko Epson Corporation, Nagano, Japan) 24 h after irradiation. The scanning direction was set to be the same for all films. For the scanned film data, the degree of blackening (*netOD*) of the film was calculated as follows:

(1) $$\mathrm{netOD}=\log_{10}\frac{{\mathrm{PV}}_{0}}{\mathrm{PV}},$$

where *PV* and *PV*_0_ denoted the pixel value of the film and the average of the pixel values in the unirradiated out-of-field, respectively.

For film calibration, the *netOD* within the uniform field at each depth (A-I) was obtained by averaging over the central area of 200 pixels × 236 pixels (33.87 mm × 40 mm). Defining *f* as the dose conversion coefficient for linearly converting the *netOD* to the physical dose, the following relationship could be obtained:

(2) $${\mathrm{Dose}}_{\mathrm{film}}=f\times netOD.$$

The nonlinear (quadratic) term [3], [4] was ignored because the limited use for the dose and dose range was supposed. The coefficient *f* was estimated, such that the film dose obtained by Eq. (2) closely matched the planned dose. In clinical practice, it is impossible to measure the dose in the body using an ionization chamber, and a comparative evaluation is possible only with the planned dose distribution. Therefore, after confirming that the planned and measured doses were equivalent, the film was calibrated based on the planned dose. To examine the relationship between the deviation of the film dose and LET, the dose-averaged LET at each measurement depth was estimated by the Monte Carlo simulation PHITS (ver.3.24) [18], [19], [20].

Using the obtained coefficient *f*, the planned dose, film dose, and ionization chamber dose distributions were compared. The planned depth and lateral dose distributions were output in steps of 1 mm by interpolation with the TPS. The film lateral distribution was obtained at each depth in the longitudinal direction of the couch by averaging over a central width of 200 pixels in the lateral direction. To eliminate film setup errors, the center of the lateral fall-off positions on both sides was set as the center of the irradiation field. To compare the field sizes, the width of the 50% dose levels relative to the prescribed physical dose was evaluated for the planned and film lateral dose distributions. Furthermore, considering practical use, the field sizes were also evaluated for the lateral distributions averaged over a narrower width (11 pixel < 2 mm).

## 3 Results

**Fig. 1:**
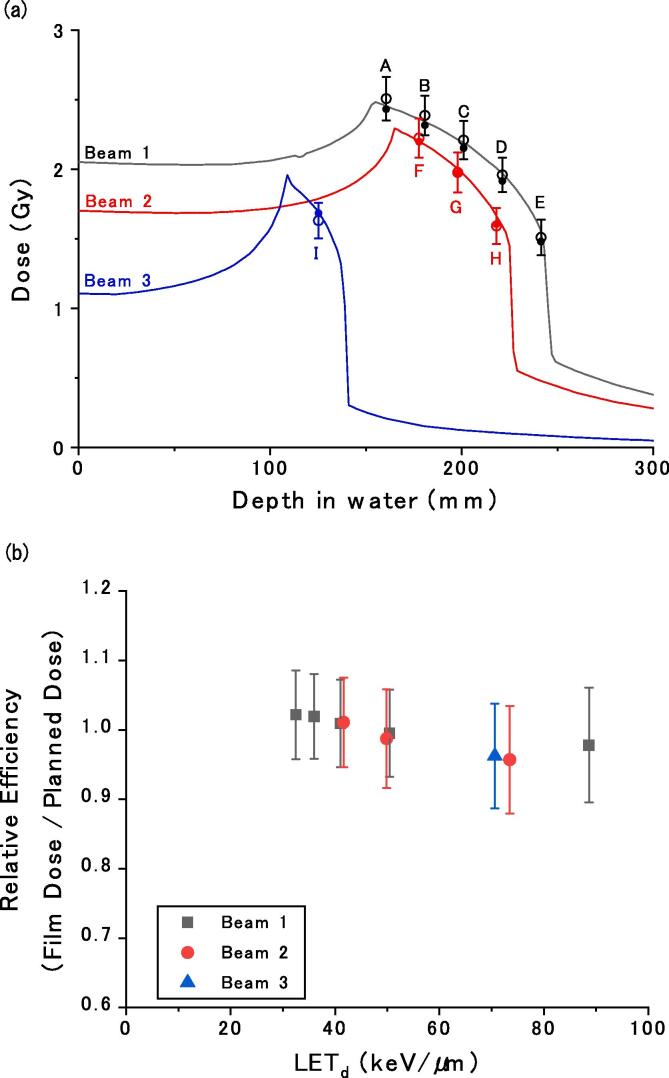
(a) Depth dose distributions of beams 1–3. The solid lines indicate planned physical dose distributions. The closed and open circles indicate the doses measured in the ionization chamber and the corresponding film doses (*Dose_film_*) obtained using Eq. (2), respectively. The letters A–I indicate the measurement depths described in Section 2.2. (b) Relative efficiency (RE) of the film response versus dose-averaged linear energy transfer (LET_d_).

The depth-dose distributions on the central axis are shown in Fig. 1(a). The coefficient *f* was determined by the least-squares fit using Eq. (2) (coefficient of determination *R*^2^ = 0.999) as 20.5 Gy. The doses measured by the ionization chamber agreed with the planned doses within 2%. The error bars represented the standard deviations that were estimated as ∼7% of the dose on average, derived from the variations in the pixel values of the films. The deviation of the film doses from the planned doses was estimated within 5% from the calibrated data points. Fig. 1(b) shows the relative film efficiency (RE), i.e., the ratio of the film dose to the planned dose versus the dose-averaged LET at each measurement depth. The RE varied from ∼1.02 to ∼0.95 as the dose-averaged LET ranged from ∼30 to ∼90 keV/μm.

**Fig. 2:**
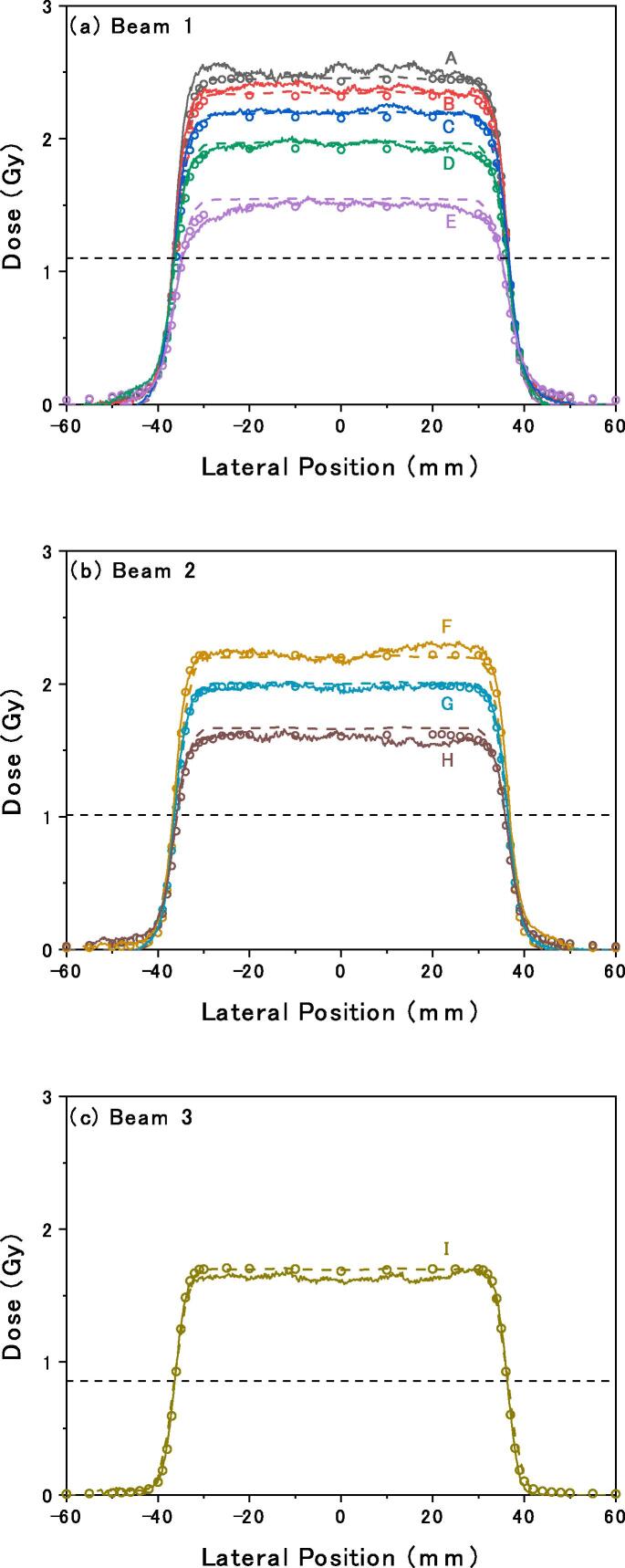
Lateral dose distributions for all measurement conditions. Dashed lines, open circles, and solid lines indicate planned dose distributions, measured dose distributions by the ionization chamber, and film dose (*Dose_film_*) distributions obtained using Eq. (2), respectively. Horizontal dashed lines indicate the 50% levels of the prescribed physical doses. The letters A–I indicate the measurement depths described in Section 2.2.

The lateral dose distributions are shown in Fig. 2. The shapes of the penumbras agreed well with one another. The absolute values of the differences in the irradiation field widths between the planned and film dose distributions for all the measurements were 0.25 ± 0.13 mm for 200-pixel width and 0.36 ± 0.36 mm for 11-pixel width.

## 4 Discussion

In this paper, we proposed a simple LET-independent film calibration for therapeutic carbon-ion beams with limited dose and LET conditions. The doses in SOBP in the range of approximately 1.5–2.5 Gy can be quantified within approximately 5% of the planned doses. The results in Fig. 1(a) show that the *netOD* to dose conversion can be sufficiently approximated by the linear relationship in Eq. (2). In addition, as derived from Fig. 2, the penumbra positions were consistent with the differences from the planned ones within 0.36 mm. Considering that the general positioning accuracy is approximately 0.5–2 mm and the actual internal motion error is greater [12], the proposed film evaluation method exhibits sufficient accuracy for confirming the irradiation field edge position for the displacement of the spacer and film because of daily organ motions.

In the film measurements shown in Fig. 1(a), the film response seems to decrease slightly relative to the planned dose toward the distal edge of the SOBP, i.e., with the increase in the dose-averaged LET. As shown in Fig. 1(b), the film response decreased by approximately 7% as the dose-averaged LET increased from ∼30 to ∼90 keV/μm. On the contrary, according to Yonai et al., regarding radiochromic EBT3 and EBT-XD films, efficiency decreases of approximately 25% were reported at the LET of 30–90 keV/μm [3]. Possible reasons for the difference could be, for example, pixel values from monochromatic reflective scans of RTQA2 films were used in this study, while Yonai et al. analyzed the red signals from colored transparent scans of EBT3 and EBT-XD films. The small LET dependence of the RTQA2 contributes to the simple calibration proposed in this study.

This is the first report of carbon-ion beam response to RTQA2 films. Further investigations with a wide range of doses and LETs are required to understand the film response more comprehensively. However, it is difficult to accurately quantify the LET distribution in the body for the actual treatment irradiation. In the proposed method that ignores the LET dependence, the accuracy of the dose estimation is limited. Nevertheless, from the present results, it is possible to quantify the dose within approximately 5% of the SOBPs of the therapeutic carbon-ion beams. This corresponds to the fact that the deviations from RE = 1.0 are within 5%, while the RE variation is ∼7% (RE = 0.95–1.02), as shown in Fig. 1(b) because the film doses are calibrated to fit the reference doses (RE = 1.0) as closely as possible. Because of variation in the pixel values of the film, when evaluated in a small area, an error of approximately 7% may be further mixed. Even in such cases, high positional accuracy is still expected, owing to the steep penumbra of carbon-ion beams. The size of the penumbra depends on the irradiation methods, e.g., the penumbra may assume the size of the pencil beam if the irradiation system is not equipped with a collimator. The dose conversion and the positional accuracy should be examined for the respective therapeutic beams adopted in the facility. Despite some deterioration in dose estimation accuracy, its positional accuracy is still sufficiently high, and therefore, it has a sufficient accuracy for confirming the irradiation field edge position from the penumbra.

In conclusion, to confirm the patient’s internal dose and irradiation field position for the restricted therapeutic carbon-ion beams, we proposed a simple LET-independent calibration method for RTQA2 films and confirmed the accuracy of the dose and irradiation field edge position. The film response exhibited quenching to some extent as the LET increased; however, the dose in the SOBP exhibited an accuracy of approximately 5% with the planned dose, and the positional accuracy of the irradiation field edge was within 0.36 mm. It exhibited sufficient accuracy as a tool for the confirmation of the penumbra position of the fields using the RTQA2 films.

## Declaration of Competing Interest

The authors declare that they have no known competing financial interests or personal relationships that could have appeared to influence the work reported in this paper.
